# Establishment of longitudinal transcranial stimulation motor evoked potentials monitoring of the forelimbs and hindlimbs in an ischemic stroke rat model

**DOI:** 10.1038/s41598-022-24835-w

**Published:** 2022-11-28

**Authors:** Masahiro Hosogai, Masaaki Takeda, Yuyo Maeda, Takahito Okazaki, Takafumi Mitsuhara, Daizo Ishii, Kiyoharu Shimizu, Masashi Kuwabara, Fumiyuki Yamasaki, Louis Yuge, Nobutaka Horie

**Affiliations:** 1 Department of Neurosurgery, Graduate School of Biomedical and Health Sciences, Hiroshima University, 1-2-3, Kasumi, Minami-Ku, Hiroshima City, Hiroshima 734-8551 Japan; 2 Division of Bio‑Environmental Adaptation Sciences, Graduate School of Biomedical and Health Sciences, Hiroshima University, Hiroshima, Japan

**Keywords:** Neuroscience, Neurology

## Abstract

Evaluation of motor function ischemic stroke rat models includes qualitative assessments such as the modified neurological severity score (mNSS). However, mNSS cannot evaluate the function of forelimbs and hindlimbs separately. We quantitatively assessed motor function in a middle cerebral artery occlusion (MCAO) rat model of ischemic stroke. We recorded transcranial stimulation motor evoked potentials (tcMEPs) from MCAO rats and measured the changes in onset latency and amplitude at the forelimbs and hindlimbs up to 28 days after stroke. All MCAO subjects showed hemiparesis. The amplitudes of tcMEPs in both fore- and hindlimbs were inversely correlated with mNSS scores, but the amplitudes in the forelimbs improved later than those in the hindlimbs. The onset latency of tcMEPs in the forelimbs and hindlimbs remained almost unchanged during the follow-up period. Our results showed the differences in tcMEPs amplitude recovery times between the forelimbs and hindlimbs after MCAO, which emphasizes the importance of separately evaluating forelimbs and hindlimbs in post-ischemic stroke models. This minimally invasive and longitudinal quantitative method could be useful for further research on diseases and neurogenesis.

## Introduction

Ischemic stroke is a major global disease with high incidence and mortality rates^1,2^. Among symptoms of ischemic stroke at major vessels, motor dysfunction is a serious presentation mainly caused by the middle cerebral artery occlusion (MCAO). In animal models of ischemic stroke, MCAO is the most common method for investigating the neurological and histological outcomes and/or pathological mechanisms of the condition^3,4^. For the evaluation of motor function in ischemic stroke rat models, including the MCAO model, “qualitative” assessments by behavioral evaluation such as the modified neurological severity score (mNSS)^5,6^, cylinder test^7–10^, and the Rotarod test^11,12^ have been used. However, “quantitative” methods have been strongly desired for an accurate evaluation of ischemic stroke rat models. It has been reported that transcranial stimulation motor evoked potentials (tcMEPs) are stable in mouse models^13^. Few reports have documented the utility of tcMEPs in rat models, some of which performed longitudinal evaluations in rat spinal cord injury models^14–16^. We previously found that tcMEPs were not reproducible for adult rats because of skull thickening during growth^17^. Therefore, we developed the technique of longitudinal tcMEP evaluations in a rat spinal cord injury model via the skull thinning method, and finally established it as a stable tcMEP recording method for the hindlimbs^17,18^.

To date, there have been no reports of long-term recordings of tcMEPs in a rat model of ischemic stroke. Moreover, there have been no reports of long-term recordings of tcMEPs for the forelimb. The assessment of forelimb function might be unnecessary for spinal cord injury models, whereas it is essential for ischemic stroke models with forelimb and hindlimb hemiparesis. We applied our skull thinning method^17^ in an MCAO rat model to determine the longitudinal quantitative evaluation of motor function by tcMEPs in the fore and hindlimbs. Here, we established the stable tcMEPs method in the MCAO rat model.

## Results

### Electrophysiological recordings

**Figure 1:**
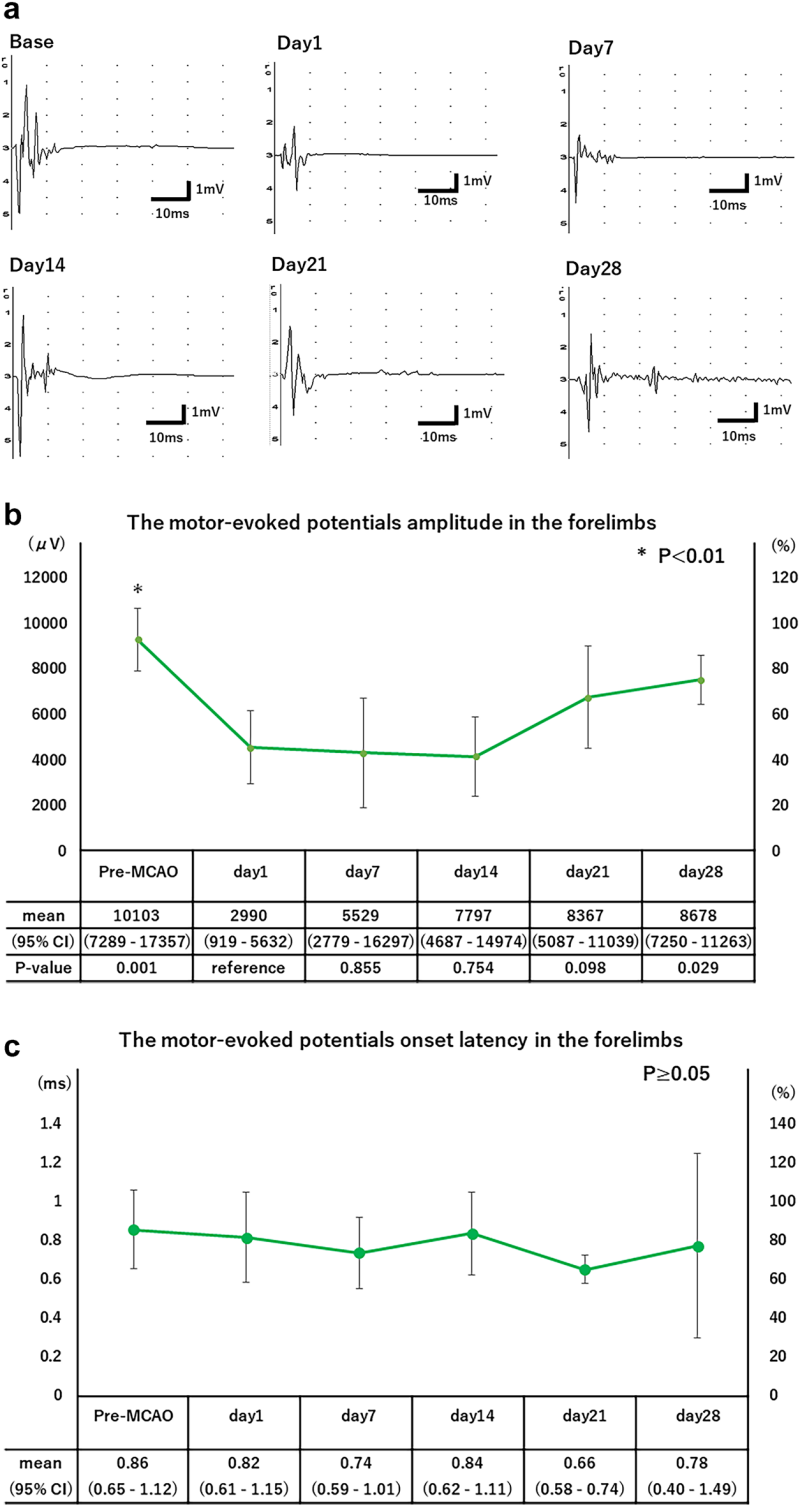
(**a**) Representative recordings of tcMEPs in the forelimbs of rats were plotted before and at 1, 7, 14, 21, and 28 days after the MCAO procedure. (**b**) Time course of the motor evoked potentials amplitude in the forelimbs. The left axis of the table shows the amplitude scale, and the right axis shows the percentage of the amplitude compared to the baseline value. Repeated measures analysis of variance was performed on the weekly measurements. Student-t tests with Bonferroni correction were performed for outcomes with significant one-way analysis of variance. *P < 0.01. (**c**) Time course of the motor evoked potentials onset latency in the forelimbs. The left axis of the table shows the onset latency scale, and the right axis shows the percentage of the onset latency compared to the baseline value. Repeated measures analysis of variance was performed on the weekly measurements.

Representative recordings of tcMEPs in the forelimbs of rats plotted before and at 1, 7, 14, 21, and 28 days after the MCAO procedure are shown in Fig. 1a. The time course of tcMEPs amplitude in the forelimbs is shown in Fig. 1b. The amplitude in the forelimbs decreased on day 1 and did not recover until day 14 after which it increased over time, changing significantly between recording dates over the postoperative course (p < 0.05, one-way ANOVA). The amplitude in the forelimbs on day 1 were significantly different from those on day 0 (p < 0.01, Student t-test with Bonferroni correction). The amplitude in the forelimbs was less than 50% of baseline between day 1 and day 14, and then recovered to 81.3% of baseline on day 28 after ischemic stroke.

The time course of tcMEPs onset latency in the forelimbs is shown in Fig. 1c. The tcMEPs onset latency in the forelimbs did not change significantly during the follow-up period (p ≥ 0.05, one-way ANOVA).

**Figure 2:**
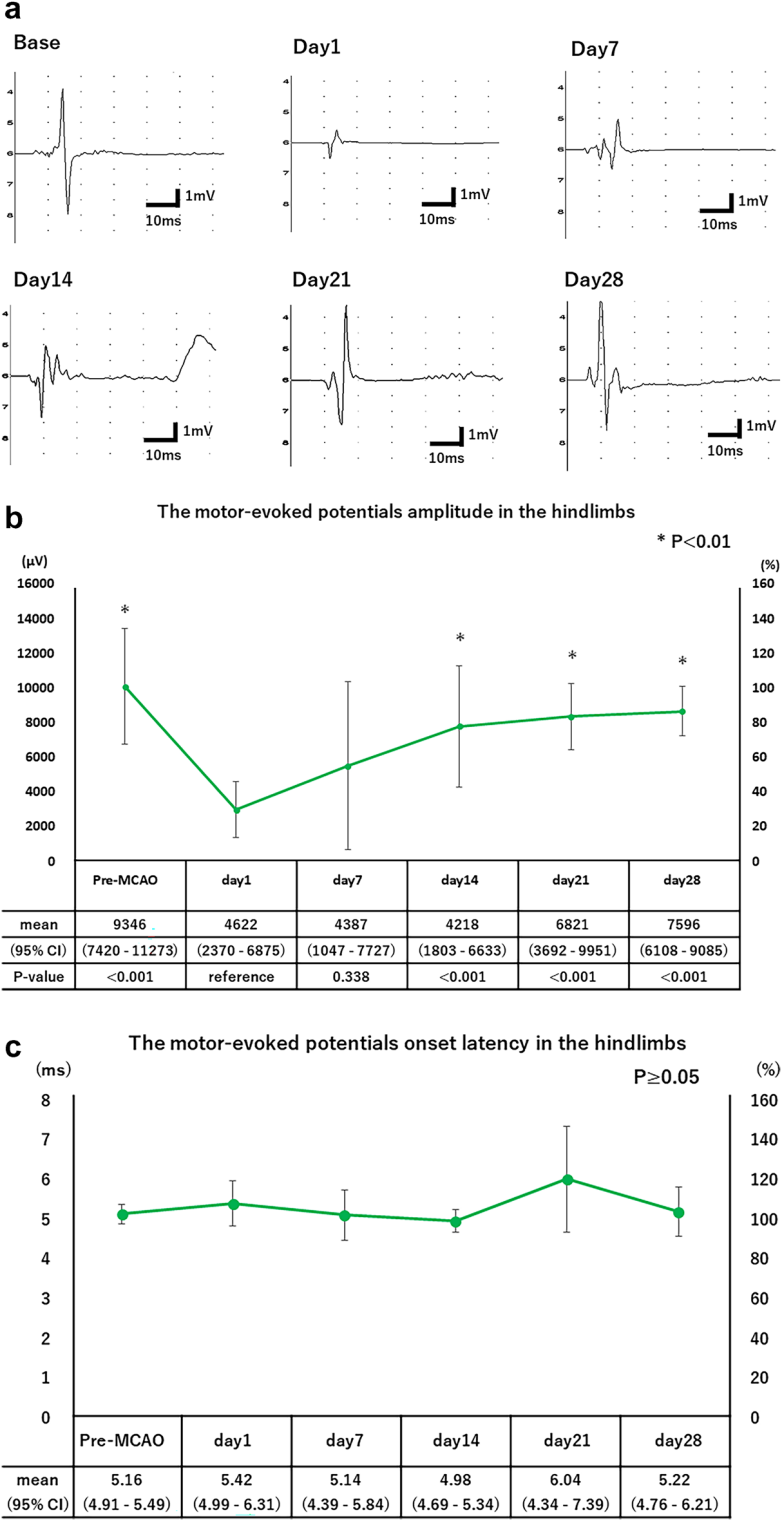
(**a**) Representative recordings of tcMEPs in the hindlimbs of rats were plotted before and at 1, 7, 14, 21, and 28 days after the MCAO procedure. (**b**) Time course of the motor evoked potentials amplitude in the hindlimbs. The left axis of the table shows the amplitude scale, and the right axis shows the percentage of the amplitude compared to the baseline value. Repeated measures analysis of variance was performed on the weekly measurements. Student-t tests with Bonferroni correction were performed for outcomes with significant one-way analysis of variance. *P < 0.01. (**c**) Time course of the motor evoked potentials onset latency in the hindlimbs. The left axis of the table shows the onset latency scale, and the right axis shows the percentage of the amplitude compared to the baseline value. Repeated measures analysis of variance was performed on the weekly measurements.

Representative recordings of tcMEPs in the hindlimbs of rats before and at 1, 7, 14, 21, and 28 days after the MCAO procedure are shown in Fig. 2a. The time course of tcMEPs amplitude in the hindlimbs is shown in Fig. 2b. The amplitude in the hindlimbs was lowest on day 1 and then increased with time, changing significantly between recording dates after the MCAO procedure (p < 0.01, one-way ANOVA). The amplitude in the hindlimbs on day 1 were significantly different from those on days 0, 14, 21, and 28 (p < 0.01, Student t-test with Bonferroni correction). The amplitude in the hindlimbs was lowest on day 1 (29.6% of baseline) and recovered to 85.9% of baseline on day 28. The time course of tcMEPs onset latency in the hindlimbs are shown in Fig. 2c. The tcMEP onset latency in the hindlimbs did not change significantly during the follow-up period (p ≥ 0.05, one-way ANOVA).

### Neurological functional recovery in the MCAO rat model

**Figure 3:**
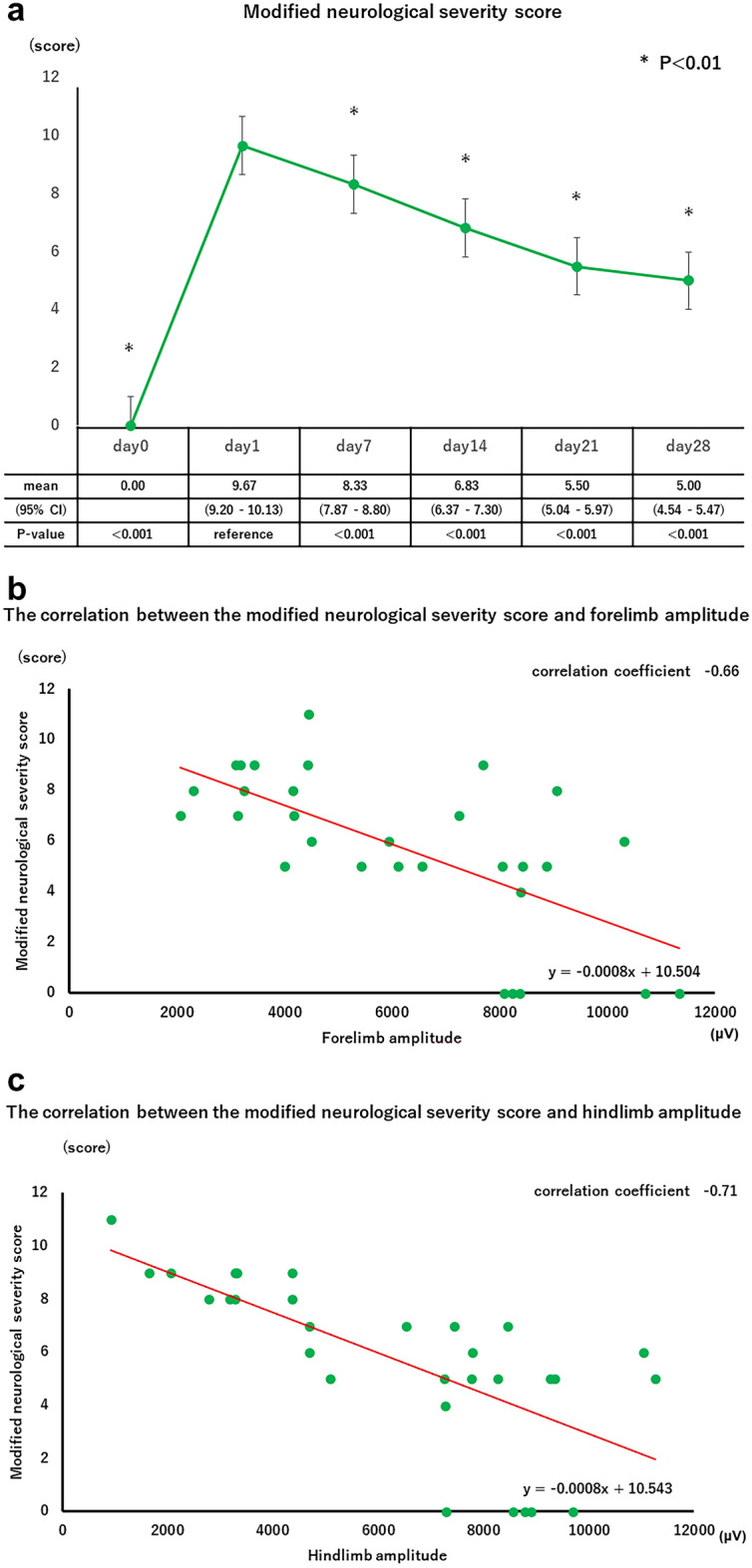
(**a**) Time course of mNSS after MCAO procedure. Repeated measures analysis of variance was performed on the weekly measurements. Student-t tests with Bonferroni correction were performed for outcomes with significant one-way analysis of variance. *P < 0.01. (**b**) Bivariate analyses of mNSS scores and the amplitudes of tcMEPs in forelimbs. (**c**) Bivariate analyses of mNSS scores and the amplitudes of tcMEPs in hindlimbs.

We assessed neurological function after the MCAO procedure using mNSS. After the MCAO procedure, all rats had left incomplete hemiparesis. The time course of the mNSS score is shown in Fig. 3a. The mNSS score was highest on day 1 and improved over time, changing significantly between recording dates after MCAO procedure (p < 0.01, one-way ANOVA). The mNSS score on day 1 were significantly different from those on days 0, 7, 14, 21, and 28 (p < 0.01, Student t-test with Bonferroni correction). Both the mNSS score and tcMEPs hindlimb amplitude improved over time after day 1, whereas the timing of improvement in tcMEPs forelimb amplitude was later than that of the mNSS score. Bivariate analyses of mNSS scores and the amplitudes of tcMEPs in both fore- and hindlimbs are shown in Fig. 3b,c. Pearson's correlation coefficients were − 0.66 and − 0.71, respectively, indicating a negative correlation between mNSS scores and the amplitudes of tcMEPs in both fore- and hindlimbs.

### Histological analysis

**Figure 4:**
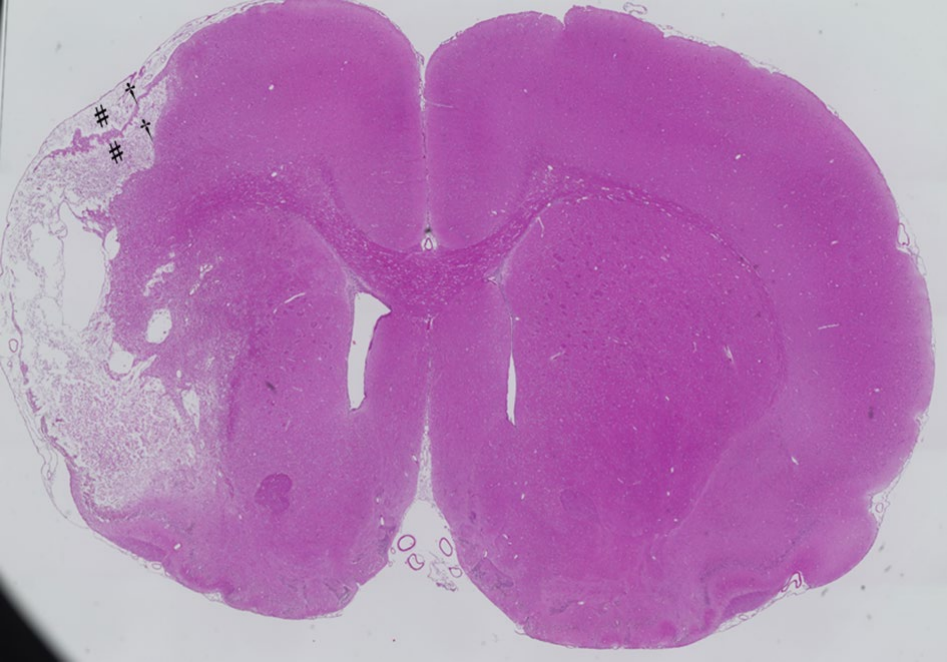
Hematoxylin and eosin (H&E) staining 28 days after middle cerebral artery occlusion. The H&E-stained segments show cerebral infarction in the middle cerebral artery region, ^#^indicates forelimb sensory-motor overlap area and ^†^indicates hindlimb sensory-motor overlap area. No brain contusion was observed by bone shaving or electrical stimulation.

The area of cerebral infarction after the MCAO procedure was evaluated histologically using hematoxylin and eosin staining. As shown in Fig. 4, the cerebral infarcts were observed in the reflux area of the middle cerebral artery, which included the sensory-motor overlap area of the forelimb at the infarction core and the hindlimb at the infarction marginal area^19^. In addition, Fig. 4 shows a section of an area where skull drilling was performed and electrical stimulation for tcMEPs recording. This section shows no brain contusion due to skull drilling nor degeneration of the brain parenchyma due to electrical stimulation.

## Discussion

In this study, we established a method for recording tcMEPs longitudinally up to 28 days after a cerebrovascular incident in an ischemic stroke rat model in both hindlimbs and forelimbs. There has been no report of long-term recordings of tcMEPs in an MCAO rat model. One study which achieved short-term recordings^20^. In that report, tcMEPs recording were performed by using stainless steel screw needle electrodes continuously implanted in the skull^20^. However, this method was highly invasive and carried a high risk of infection, making a long-term recording of tcMEPs difficult. For the long-term recording of tcMEPs in rat models, it is necessary to reduce the level of invasiveness. Our method of using percutaneous needle electrodes for stimulation and recording is a minimally invasive and simple procedure that allows repeated electrophysiological examinations in the same animal. In the present study, tcMEPs could be recorded over a longer period because continuous electrode placement was unnecessary, and the procedure was minimally invasive since the electric needle was only inserted for each recording.

The tcMEPs amplitude in the hindlimbs was lowest on day 1 and then recovered over time to 85.9% of the baseline on day 28. Conversely, the amplitude in the forelimbs did not begin to recover until day 14, and then it improved to 81.3% of the baseline on day 28. A previous report using the cylinder test in cortical and striatal cerebral infarction rat models showed only a slight improvement in forelimb function up to 14 days after onset, and then showed a moderate improvement until day 28^7^. Our quantitative evaluation of the forelimb amplitude of tcMEPs was consistent with their qualitative results obtained from the cylinder test. The qualitative mNSS method for assessing locomotor function showed moderate improvement on day 7 and then continuous improvement over time. We found that our quantitative evaluation of the hindlimb amplitude at tcMEPs was consistent with the results of mNSS. We speculated that the delay of forelimb compared to the hindlimb improvement might be caused by the area of MCAO infarction; the forelimb area was at the core, and the hindlimb area was at the marginal section of the infarction. Future studies should explore mechanisms underlying the difference between the improvement of forelimb and hindlimb function with the evaluation of not only mNSS, but also with the cylinder test.

To date, there have been no reports of long-term recordings of tcMEPs in the forelimbs in ischemic stroke models. One reason may be that tcMEPs of the forelimbs are difficult to evoke because the motor area of the forelimb is located more outward than that of the hindlimbs, making it more difficult for the electrical stimulation to be transmitted. Our method of tcMEPs via thinning the skull could reduce electrical resistance and overcome the challenge of electrical transmission. With this method, we were able to evaluate the motor function of not only the hindlimbs but also the forelimbs in an ischemic stroke rat model. Long-term recordings allowed us to evaluate the changes in motor function in detail.

The onset latency of tcMEPs in both the fore- and hindlimbs did not change significantly over the 28 day follow-up period. The onset latency of muscle response to cortical stimulation represent the sum of central and peripheral conduction times, and central motor conduction time is calculated by subtracting the peripheral conduction time from the MEPs onset latency elicited by motor cortical stimulation^21^. When patients' corticospinal tracts are damaged, axonal degeneration is reported to occur about 1 month after onset, prolonging central motor transmission time^21,22^. In contrast, in rats, axonal degeneration of the corticospinal tract is reported to occur within 1 to 3 weeks after ischemic stroke, but it is unclear whether the central motor conduction time is prolonged as well^23^. In addition, central motor conduction time has been reported to correlate with the severity of neurological defects^24^. Therefore, in the present study, the onset latency may have remained almost unchanged because of motor function improved over time after an ischemic stroke. On the other hand, there is a report that the onset latency of tcMEPs in both the fore- and hindlimbs was prolonged in rats 7 days after ischemic stroke, which differs from our results^20^. However, it has also been reported that the degree of cerebral infarction and cerebral edema affect the central motor conduction time^25^, and this difference may be due to differences in the severity of the ischemic stroke models.

Behavior rating scales such as the mNSS have the advantage of being easy to measure, but they are qualitative. In contrast, tcMEPs are quantitative and can more accurately assess the motor function of rats. Furthermore, our results show that forelimb and hindlimb motor functions can be evaluated separately. This minimally invasive and longitudinal quantitative method could be useful for further research, such as studies investigating the effectiveness of cell therapies and rehabilitation in cerebral infarction rat models.

In conclusion, we established a stable tcMEPs method for the longitudinal assessment of motor function at not only the hindlimbs, but also the forelimbs in a MCAO rat model. We showed the difference in tcMEPs amplitude recovery timing between the forelimbs and the hindlimbs after MCAO. Quantitative assessment of forelimb and hindlimb motor function in rats appears to be essential for understanding the mechanisms of brain damage in stroke.

## Methods

### Ethics statement

All methods have been reported in accordance with the ARRIVE guidelines (https://arriveguidelines.org) and the American Veterinary Medical Association (AVMA) Guidelines for the Euthanasia of Animals (2020). All methods were performed in accordance with the relevant guidelines and regulations. All study protocols were approved by the Animal Testing Committee Guidelines of Hiroshima University, Japan, and conducted under the authority of the Project License (A19-164).

### Electrophysiological recordings

**Figure 5:**
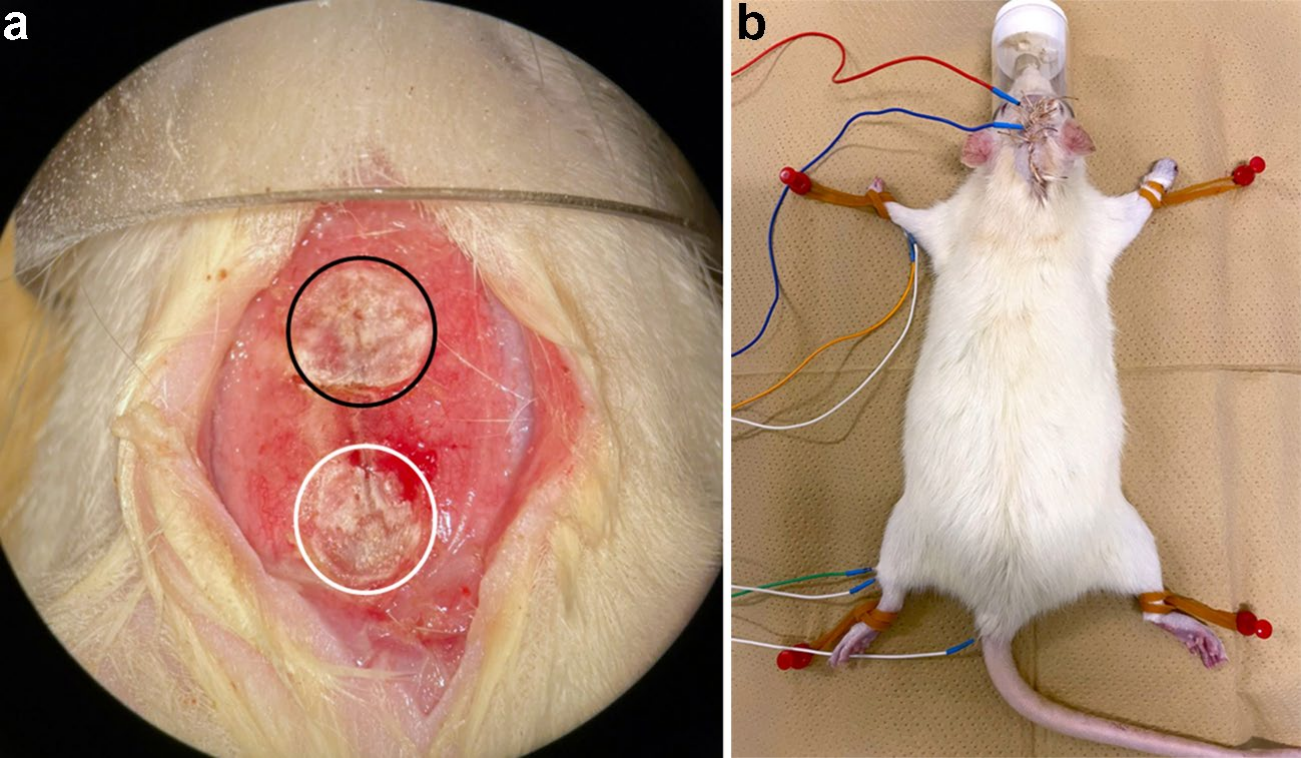
(**a**) The procedure of skull thinning. The outer layer and diploe of the skull were resected with the size of 5 mm diameter at both bregma and lambda by the diamond drill. The black circle indicates the bregma after bone thinning, and the white circle indicates the lambda after bone thinning. The anode was placed at lambda and the cathode at bregma. (**b**) The red electrode is an anode which is inserted into the bregma position. The blue electrode is a cathode which is inserted into the lambda position. Two electrodes were inserted in the left forelimb's extensor muscles and the left hindlimb's quadriceps muscles, respectively, and the ground wire was inserted into the tail. Transcranial electrical stimulation motor evoked potentials were recorded under general anesthesia.

Five female adults Sprague–Dawley rats (Charles River, Kanagawa, Japan) with a bodyweight range of 250–300 g were housed at a room temperature of 23 ± 1 °C, humidity of 50 ± 10%, and a light/dark cycle of 12 h with ad libitum access to food and water. Referring to previously published reports that have recorded tcMEPs in a similar number of animals^26^, we deemed our sample size sufficient to evaluate our model. Inhalation anesthesia was induced with 1.5% isoflurane, and during the procedure, body temperature was maintained at 37 ± 0.5 °C using a thermostat-controlled heating pad (Bio Research Center Co., Ltd., Nagoya, Aichi, Japan). After the linear skin incision, the skull was exposed, and then the skull was thinly shaved (total area diameter = 5 mm) with a diamond drill at the bregma and lambda positions as previously reported (Fig. 5a)^17,18^. This skull thinning technique created a low electrical resistance field for optimal electrical stimulation of the motor cortex. Transcranial electrical stimulation was performed through the thinned skull position using needle electrodes (Natus Medical Inc., Middleton, WI, USA). The anode was installed at the lambda position and the cathode at the bregma position. Transcranial electrical stimulation was performed by administering four stimulus trains over a total duration of 0.5 ms with supramaximal stimulation. The strength of stimulation for each rat was decided by supramaximal stimulation before the MCAO procedure, and the stimulation strength was kept constant during the time course of tcMEPs experiments for each subject. The supramaximal stimulation intensity was defined as a value that is at least 20% higher than the stimulus intensity at which the maximum amplitude of the compound muscle action potential can be recorded. The stimulus intensity of MCAO models for the forelimb and hindlimb was 34 V and 66 V, respectively. We recorded tcMEPs at least five times per session and the suitable waveform was selected after confirming that stable waveforms (latency and amplitude were almost the same) were recorded. To record tcMEPs, two needle electrodes were inserted in the forelimb's extensor muscles and the hindlimb's quadricep muscles, respectively, and the ground wire was inserted into the tail (Fig. 5b). Stimulation and recording were performed using Endeavor CR (Nicolet Biomedical, Madison, WI, USA), and the needles for transcranial electrical stimulation were inserted percutaneously before every recording and then removed after every recording. The onset latency and amplitude of tcMEPs were recorded before and 1, 7, 14, 21, and 28 days after the MCAO procedure.

### Surgical procedure for MCAO and the neurological function test

After measuring the reference value of tcMEPs, the transient MCAO procedure was performed by an intraluminal thread occlusion method, as previously described^27^. The right common carotid artery, external carotid artery, internal carotid artery, and pterygopalatine artery were exposed under isoflurane anesthesia using a microscope. A 4–0 nylon monofilament suture coated with silicone (403745PK10. Doccol Co., Sharon, MA, USA) was inserted from the cut stump of the external carotid artery toward the internal carotid artery until it blocked the origin of the right MCA. Following one and half hours of occlusion, the thread was retracted for reperfusion. Body temperature was maintained at 37 ± 0.5 °C as described above during the procedure.

Neurological function was assessed using mNSS, which is a composite score of motor, sensory, balance, and reflex responses on a scale from 0, indicating normal, to 18, indicating major impairment^28^. The mNSS tests were performed before and on days 1, 7, 14, 21, and 28 after MCAO. The mNSS scores of all rats on day 1 after the MCAO procedure were between 9 and 11, and we used these rats in this study.

### Statistical analysis

Continuous variables were presented as the mean (standard deviation) and 95% confidence interval (CI). Weekly continuous variable comparisons were performed using repeated measures one-way analysis of variance (ANOVA). Student-t tests with Bonferroni correction were performed for outcomes in which one-way ANOVA were significant. P-values < 0.05 were considered statistically significant in one-way ANOVA. P-values < 0.01 were considered statistically significant in Student-t tests with Bonferroni correction. Bivariate analysis was evaluated by correlation coefficients. Statistical analyses were performed using JMP® Pro version 16 (SAS Institute, Cary, NC, USA).

## Data Availability

The raw data supporting the findings of this study are displayed in Figs. 1, 2, 3, 4, and 5. The authors declare that all data supporting the findings of this study are available within the paper.
